# Prophylactic treatment can modify vascular risk biomarkers in high-frequency episodic and chronic migraine patients: a pilot study

**DOI:** 10.1038/s41598-023-44522-8

**Published:** 2023-11-08

**Authors:** Cristina González Mingot, Sonia Santos Lasaosa, Laura Colàs Campàs, Laura Chilangua Canaval, Anna Gil Sánchez, Luis Brieva Ruiz, María Cristina Marzo Alonso, Silvia Peralta Moncusi, Joan Valls Marsal, Serafí Cambray Carner, Francisco Purroy García

**Affiliations:** 1 Neuroimmunology Group, Department of Medicine, University of Lleida (UdL)-IRBLleida, Alcalde Rovira Roure 80, 25198 Lleida, Spain; 2 Neurology Service of Hospital Arnau de Vilanova of Lleida, Lleida, Spain; 3 Neurology Service of Hospital Clínico Lozano Blesa of Zaragoza, Zaragoza, Spain; 4 Biomedical Research Institute of Lleida, Lleida, Spain; 5 Neurology Service of Hospital Son Espases, Palma, Illes Balears Spain; 6 Hematology Service of Hospital Arnau de Vilanova of Lleida, Lleida, Spain

**Keywords:** Neuroscience, Biomarkers, Neurology

## Abstract

To evaluate whether preventive treatment can modify endothelial and oxidative biomarkers of vascular disease risk in patients with high-frequency episodic and chronic migraine. In this observational, prospective pilot study, 88 prophylactic treatment-naïve patients with episodic and chronic migraine and 56 healthy sex/age matched controls underwent ultrasonography exams and blood tests at baseline, and again in the migraine patients after 3 months’ treatment with metoprolol or topiramate. Biomarkers for endothelial function and oxidative stress were analyzed. At baseline, patients with migraine in the low-frequency episodic group had differences exclusively in nitrates 17.6 versus 27.33 µM; *p* = 0.046 compared to the controls. However, when comparing the group comprised of patients with high-frequency episodic migraine and chronic migraine versus controls, statistically significant differences appeared in hsCRP 2.68 versus 1.64 mg/dL; *p* = 0.049, vWF antigen (133% vs. 110%; *p* = 0.020, vWF activity (111% vs. 90%; *p* = 0.010) and isoprostane levels (181 vs. 238 µM; *p* = 0.05). Only in the chronic migraine subgroup did we found statistically significant differences in CIMT (0.60 vs. 0.54 mm; *p* = 0.042) which were significantly greater than in the controls. After treatment, patients who respond to preventive treatment exhibited significantly higher levels of nitrates (24.2–13.8 µM; *p* = 0.022) and nitrites (10.4–3.43 µM; *p* = 0.002) compared than non-responders. Moreover, biomarker levels improved in treatment-responsive patients with migraine; hsCRP levels decreased from 2.54 to 1.69 mg/dL (*p* < 0.05), vWF activity levels decreased from 124 to 103 IU/dL (*p* = 0.003) and prothrombin activity decreased from 1.01 to 0.93 (*p* = 0.01). These differences were also observed in the high-frequency and chronic migraine subgroup and reach statistical significance in the case of hsCRP, which decreased from 2.12 to 0.83 mg/dL (*p* = 0.048). Patients with migraines have differences in biomarker levels compared to controls, suggesting endothelial and oxidative dysfunction. The greatest differences in biomarker levels compared to controls are observed in migraine patients in the high-frequency and chronic migraine subgroups. Based on our results, preventive treatment is capable of modifying markers of endothelial dysfunction and oxidative stress in migraine patients, even in cases of chronic and high-frequency migraine.

## Introduction

Migraine is a common, debilitating type of headache and the sixth most prevalent disorder globally^1^. It usually begins episodically, with the nervous system returning to a normal or premorbid functional state between attacks. However, approximately 2.5% of patients with episodic migraine (EM) experience transformation to Chronic migraine (CM)^2^.

CM is a complication of migraine that is defined by the presence of ≥ 15 headache days per month for at least 3 months, with at least 8 of those days meeting criteria for migraine, in the absence of medication overuse and other causes to which the headache could be attributed^3^. The pathophysiology of CM and the mechanisms responsible for migraine chronification are not fully understood, although atypical pain processing during migraine episodes, central sensitization, cortical hyperexcitability, and neurogenic inflammation have been proposed as possible causes^4^.

Prophylactic drugs are utilized in order to reduce the severity, duration, and frequency of migraines. First line prophylactic therapies include ß-adrenergic blockers (such as metoprolol), anticonvulsants (topiramate), and antidepressants (such as amitriptyline). Botulinum toxin A (BoNT/A) and calcitonin gene-related peptide monoclonal antibodies are indicated in high frequency and CM cases^5^.

Migraine has been considered a neurovascular disorder since the phenomena of cortical spreading depression and neurogenic inflammation were described in its pathophysiology^6^. Two major types of migraine exist: migraine with aura (MA) and migraine without aura (MO). The main difference between MA and MO is that MA is characterized by transient focal neurologic symptoms that usually precede or sometimes accompany the headache^7^.

The migraine aura plays a key role in connecting migraine with stroke risk, since the increase in the relative risk of ischaemic stroke is doubled only in MA, in most studies. Cortical spreading depression, the underlying electrophysiological mechanism of migraine aura is a wave of increased electrocortical activity and vasodilation, followed by sustained decreased activity and prolonged vasoconstriction resulting in a 20–30% decrease in cerebral blood flow. Under normal circumstances, the reduction of cerebral blood flow is insufficient to cause cell damage. However, in rare instances, the hypoperfusion might become severe enough to cause ischemia^8^.

Endothelial dysfunction, characterized by impaired endothelial activation and vascular response, is mediated by oxidative stress^9^. There is growing evidence linking endothelial dysfunction to migraines; indeed, if migraines are considered to be a systemic vasculopathy, it is possible that endothelial dysfunction contribute to the underlying pathophysiology of migraines^10^. Migraine and particularly MA, has consistently been associated with a increased risk of experiencing ischemic strokes^10^. MA is associated with an unfavorable cardiovascular risk profile and an elevated 10-year predicted risk of coronary heart disease based on the Framingham Risk Score^8^. In addition, there is strong evidence for an association between migraine and cardiovascular diseases such as stroke^11,12^, atrial fibrillation^13^, myocardial infarction^14^ and metabolic syndrome^15^. This association is stronger in women and those with MA^13,16^, even without traditional risk factors^8^. Although the mechanism underlying the migraine-stroke association remains unknown, it could be beneficial to identify and modify vascular risk factors^8^. We have less evidence focused on chronic and high-frequency episodic migraine (HFEM)^9,17,18^. A few studies investigating the effects of prophylactic migraine treatment on oxidative stress, endothelial dysfunction, and stroke prevention exist^19–21^; but, there is no definitive evidence that controlling migraine attacks with preventive drugs reduces cardiovascular risk^8^.

In this study we aimed to evaluate whether preventive treatment can modify endothelial and oxidative biomarkers of vascular disease risk in patients with HFEM and CM. For this purpose we have investigated the effects of 3 months of prophylactic therapy with either topiramate or metoprolol on these biomarkers.

## Methods

### Patient population

This observational, multicenter prospective pilot study consecutively recruited individuals referred to the headache unit of the Hospital Universitari Arnau de Vilanova (Lleida, Spain) and Hospital Clinico Lozano Blesa (Zaragoza, Spain) between 2015 and 2018. Patients with migraine were enrolled if they had experienced four or more days of migraine per month, were between 18 and 50 years old, and had no history of prophylactic treatment use (i.e., were treatment naïve; see Additional File 1; Supplementary Table S1 for full inclusion and exclusion criteria). The diagnosis of migraine, as well as the diagnosis of episodic and chronic migraine, was made based on the International Classification for Headache Disorders-III diagnosis criteria^7^. The control group included healthy volunteers (nurses, doctors, and medical students from the Hospital Universitari Arnau de Vilanova [Lleida, Spain]) without migraine who were matched by sex and age to the migraine group. Volunteers did not receive financial compensation for participating. Participants were excluded from either group if they had cardiovascular disease (atrial fibrillation, chronic kidney disease, heart failure, coronary artery disease, stroke), cardiovascular risk factors (obesity, hypertension, diabetes mellitus, dislypemia), oncologic or inflammatory disease, were smokers, taking antioxidants, drugs with cardiovascular pleiotropic effect, or drugs acting on the arterial wall. Participants taking hormonal contraceptive pills and women who were pregnant were also excluded. This study was conducted according to the ethical principles of the World Medical Associations Declaration of Helsinki. Ethics approval was obtained from the Arnau de Vilanova of Lleida hospital’s ethics committee (approval ID: CEIC-861) and written informed consent was obtained from all participants.

T0 corresponds to the baseline visit, while T1 corresponds to the longitudinal visit conducted at 3 months after the initiation of preventive treatment. Migraine frequency at baseline (T0) was classified based on the inclusion criteria specified in Additional File 1; Supplementary Table S1.

**Figure 1:**
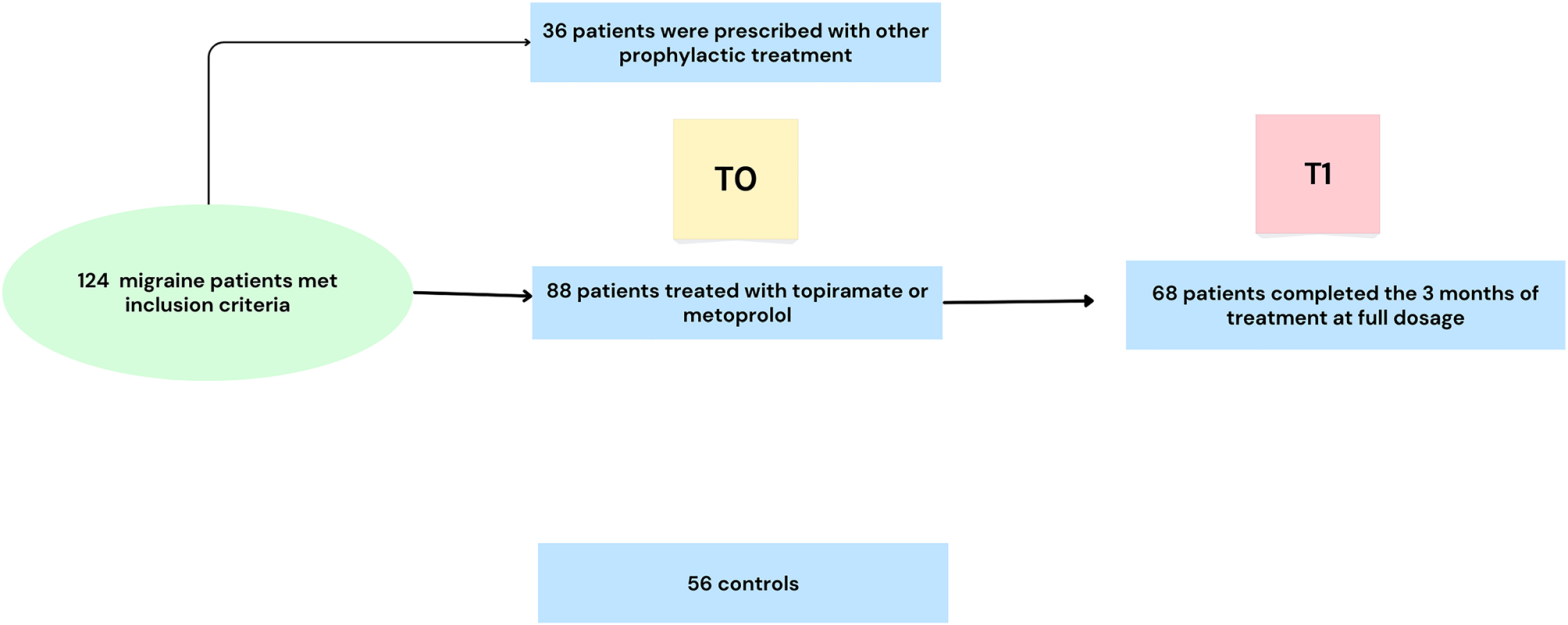
Flow chart of the study.

124 patients met the inclusion criteria. Among them, 36 patients were prescribed a different preventive treatment other than topiramate or metoprolol due to their medical history (mainly asthma, hypotension, low weight, or nephrolithiasis). There were 56 controls and 88 patients at baseline (T0). Of these, 68 completed the 3 months of treatment at full dosage, and they were included in the longitudinal visit (T1) (Fig. 1).

### Biomarkers

At T0, endothelial dysfunction (inflammatory markers and endothelium-derived vasoactive factors) and oxidative stress biomarkers were assessed in the migraine and control groups. Venous blood samples were collected, and carotid ultrasonographies were performed on both sides of the participants’ necks following the Mannheim methodology^22^. Patients in the migraine group repeated these tests 3 months (T1) after starting treatment with a migraine prophylactic drug (either topiramate or metoprolol). The decision to initiate treatment was made at the discretion of the treating clinician, independent of study investigators. Patients were presented both drug options (provided they did not present with any comorbidity that may contraindicate either treatment) and chose their preferred treatment based on dosage and possible adverse effects. All treatments were prescribed according to the local prescribing information. All investigations in migraine patients were conducted during interictal periods, 72 h after a migraine attack and use of a migraine abortive treatment (including NSAIDs).

Venous blood samples were stored in the biobank at − 80 °C and analyzed at the hospital, while oxidative stress biomarkers were assayed at the Institute of Biomedical Research of Lleida according to manufacturer instructions^23–25^. Carotid ultrasonographs were assessed, and the pulsatility index (PI) measured, by the same experienced neurologist within the neurology department.

Biomarkers for endothelial dysfunction (Additional File 2; Supplementary Table S2) included inflammatory markers and endothelium-derived vasoactive factors as high-sensitivity C-reactive protein (hsCRP), total cholesterol, high-density lipoprotein (HDL) cholesterol, low-density lipoprotein (LDL) cholesterol, triglycerides, prothrombin activity, antithrombin, fibrinogen, plasminogen, factor II, homocysteine, platelet count, von Willebrand factor (vWF) antigen and activity, carotid intima-media thickness (CIMT), pulsatility index (PI), nitrat, nitrite, and isoprostane levels. Biomarkers for oxidative stress included total oxidant status (TOS), thiol and thiobarbituric acid reactive substances (TBARS) levels.

### Treatment response

Treatment response was defined as a > 50% reduction from T0 in migraine frequency at T1.

### Statistical analysis

Continuous variables were expressed as mean ± standard deviation (SD) and categorical variables were expressed as the absolute frequency and percentage. To assess the association of different risk factors with clinical variables of interest, the Pearson chi-square test or the student’s t test were used as appropriate. To evaluate the difference between measurements from T0 to T1, a pre-test and post-test analysis was performed using a Wilcoxon signed rank test for paired non-parametric data, using all available data points for each measurement. Statistical significance was < 0.05, setting the threshold at alpha = 0.05. All statistical tests were carried out using the statistical software package R version 4.2.

### Ethics approval and consent to participate

This study was conducted according to the ethical principles of the World Medical Associations Declaration of Helsinki. Ethics approval was obtained from the hospital’s ethics committee (approval ID: CEIC-861) and written informed consent was obtained from all participants.

## Results

Overall, 144 individuals were enrolled in this study (88 with migraine and 56 controls). The majority of participants in the migraine group were female (87.5%) and their mean ± SD age was 36.9 ± 10.2 years (Table 1). The control group were matched by sex and age (82.1% female, mean age 35.9 ± 11.2 years).

**Table 1:** Baseline characteristics in patients with migraine.

	Migraine (n = 88)	Controls (n = 56)
Age, years, mean ± SD	36.9 ± 10.2	35.9 ± 11.2
Gender, n (%)		
Female	77 (87.5)	79 (82.1)
Male	11 (12.5)	10 (17.9)
Migraine type, n (%)		
With aura	32 (36.4)	–
Without aura	56 (63.6)	–
Migraine frequency, headache days/month, mean ± SD	9.3 ± 5.8	–
Migraine frequency, n (%)		
Low (4–7 headache days/month)	33 (37.5)	–
High (8–14 headache days/month)	33 (37.5)	–
Chronic (≥ 15 headache days/month, of which ≥ 8 days are days with a migraine)	22 (23.9)	–

*SD* standard deviation.

At T0, 37.5% of the patients with migraine had low-frequency EM (LFEM), 62.5% experienced HFEM and CM (Table 1). Overall, 20 patients from the migraine group were excluded from the evaluation at T1 either because they did not receive full-dose treatment or they were lost to follow-up. Moreover, no missing values were observed for baseline characteristics; however for biomarker measurements, missing data presence ranged from 1 to 59% (Supplementary Material Table S1).

### Biomarkers at T0

#### Biomarkers in patients with migraine versus controls

HsCRP was significantly higher in patients with migraines (2.55 vs. 1.64 mg/dL; *p* = 0.025) while HDL cholesterol (61.7 vs. 66.8 mg/dL; *p* = 0.048), nitrate (19.4 vs. 27.3 µM; *p* = 0.037) and isoprostane levels (181 vs. 238 µM; *p* = 0.036; Table 2) were significantly lower.

**Table 2:** Biomarkers at baseline in patients with migraine and controls.

	Patients with migraine (n = 88)	Controls (n = 56)	*p* value
Endothelial dysfunction biomarkers			
Total cholesterol, mg/dL	189 ± 33.2	187 ± 32.4	0.669
HDL cholesterol, mg/dL	61.7 ± 14.7	66.8 ± 14.9	**0.048**
LDL cholesterol, mg/dL	109 ± 28.0	103 ± 29.2	0.272
Triglycerides, mg/dL	94.3 ± 41.4	84.1 ± 35.8	0.119
hsCRP, mg/dL	2.55 ± 2.83	1.64 ± 1.73	**0.025**
Prothrombin activity, %	0.99 ± 0.08	2.85 ± 13.5	0.317
Antithrombin,%	104 ± 11.3	101 ± 9.17	0.215
Fibrinogen, mg/dL	3.94 ± 0.67	3.91 ± 0.78	0.827
Plasminogen,%	110 ± 17.4	106 ± 15.8	0.281
Factor II%	106 ± 15.5	112 ± 17.5	0.144
Homocysteine, µmol/L	7.66 ± 2.76	8.41 ± 3.03	0.156
Platelet count, mL/mm^3^	269 ± 75	248 ± 64	0.095
vWF antigen, IU/dL	121 ± 40.8	110 ± 40.6	0.244
vWF activity, %	102.0 ± 34.7	90 ± 34.7	0.063
CIMT, mm	0.55 ± 0.09	0.54 ± 0.07	0.309
PI, cm/s	0.79 ± 0.16	0.84 ± 0.16	0.184
Nitrate, µM	19.4 ± 17.5	27.3 ± 19.4	**0.037**
Nitrite, µM	11.1 ± 9.35	10.2 ± 7.76	0.598
Isoprostane, µM	181 ± 134	238 ± 137	**0.036**
Oxidative stress biomarkers			
TOS, µmol/L	846 ± 653	784 ± 652	0.647
Thiol, nM	216.511 ± 33.763	221.839 ± 29.888	0.396
TBARS, µM	1.16 ± 0.52	1.11 ± 0.48	0.626

Significant values are in [bold].

All values are mean ± standard deviation.

*CIMT* carotid intima-media thickness, *hsCRP* high-sensitivity C-reactive protein, *HDL* high-density lipoprotein, *LDL* low-density lipoprotein, *PI* pulsatility index, *TBARS* thiobarbituric acid reactive substances, *TOS* total oxidant status, *vWF* von Willebrand factor.

Although patients with migraines had higher levels of endothelial dysfunction biomarkers CIMT, total cholesterol, LDL cholesterol, triglycerides, platelets, vWF antigen, and vWF activity compared to matched controls (Table 2); these differences did not reach statistical significance. Significant differences were also not observed between the two groups with other biomarkers of oxidative stress.

#### Biomarkers by migraine frequency

##### LFEM group (4–7 migraine days/month)

When we subdivide the patients with migraine according to their frequency and compare them with controls, we observed statistically significant differences in the LFEM group (4–8 migraine days/month) exclusively in nitrates 17.6 versus 27.33 µM; *p* = 0.046 ).

##### HFEM plus CM group (≥ 8 days migraine days/month)

However, when comparing the HFEM (8–14 migraine days/month) and CM group (≥ 15 headache days/month, of which ≥ 8 days are days with a migraine), in other terms, the subgroup of patients with ≥ 8 days of headache per month versus controls, statistically significant differences also appear in hsCRP 2.68 versus 1.64 mg/dL; *p* = 0.049, vWF antigen (133% vs. 110%; *p* = 0.020, and vWF activity (111% vs. 90%; *p* = 0.010) which were significantly greater in the patients than in the controls. While isoprostane levels (181 vs. 238 µM; *p* = 0.049) in this subgroup with ≥ 8 days of headache per month were significantly lower than in the controls.

##### CM group (of ≥ 15 headache days per month for at least 3 months, with at least 8 of those days meeting criteria for migraine)

Only in the CM subgroup did we find statistically significant differences in CIMT (0.60 vs. 0.54 mm; *p* = 0.042) which were significantly greater than in the controls.

No significant differences were observed between the remaining disease frequency subgroups and controls.

**Figure 2:**
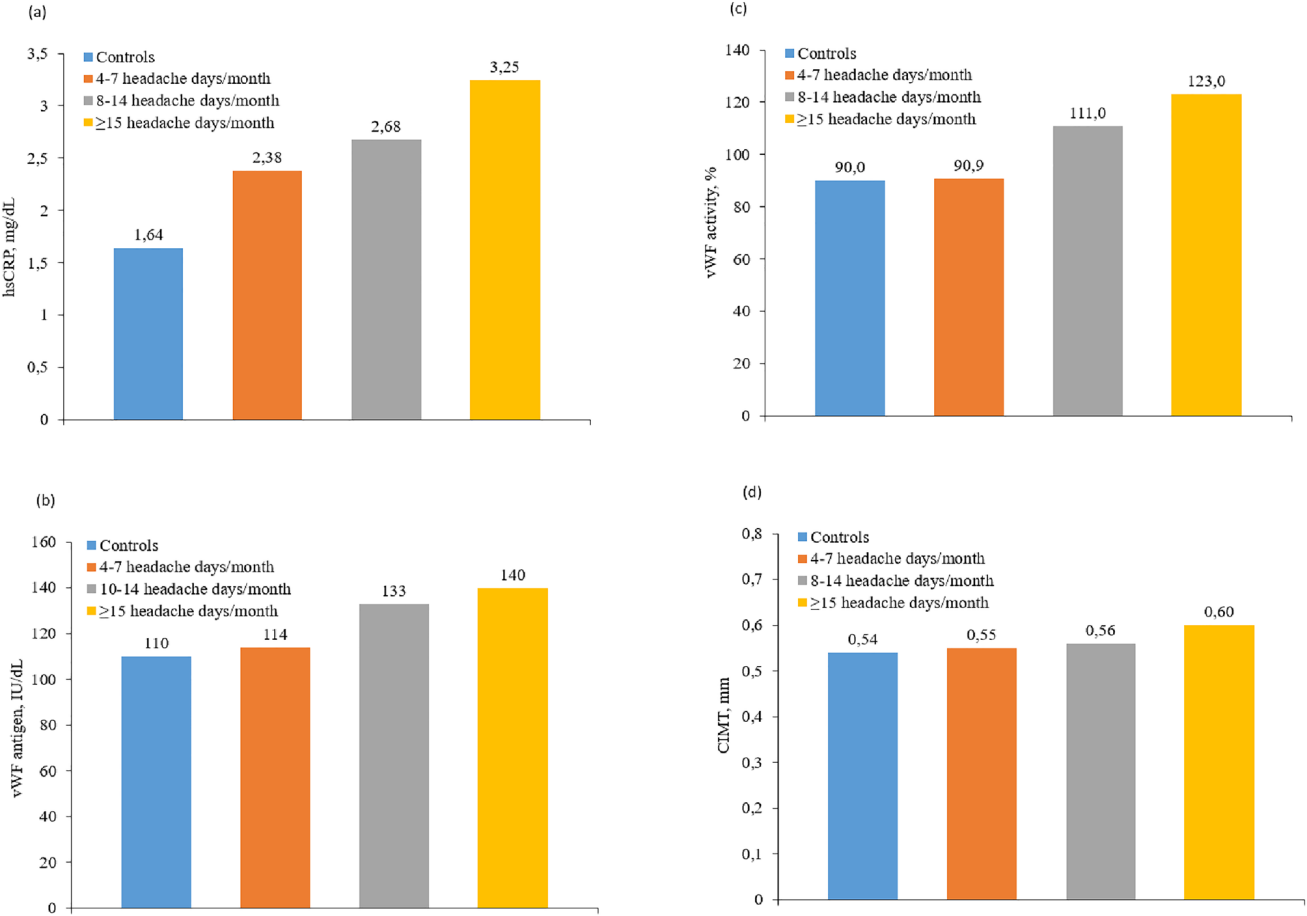
(**a**) hsCRP, (**b**) vWF antigen, (**c**) vWF activity, (**d**) CIMT at baseline by migraine severity. A positive correlation was found between the inflammatory and endothelial dysfunction biomarkers: CIMT (*p* = 0.024), vWF antigen (*p* = 0.045), and vWF activity (*p* = 0.013). In the case of HsCRP, we observe a trend, but it does not reach statistical significance (*p* > 0.05). Low [4–7 headache days/month], high [8–14 headache days/month] and chronic [≥ 15 headache days/month of which ≥ 8 days are days with a migraine]. *CIMT* carotid intima-media thickness, *hsCRP* high-sensitivity C-reactive protein, *vWF* von Willebrand factor.

**Figure 3:**
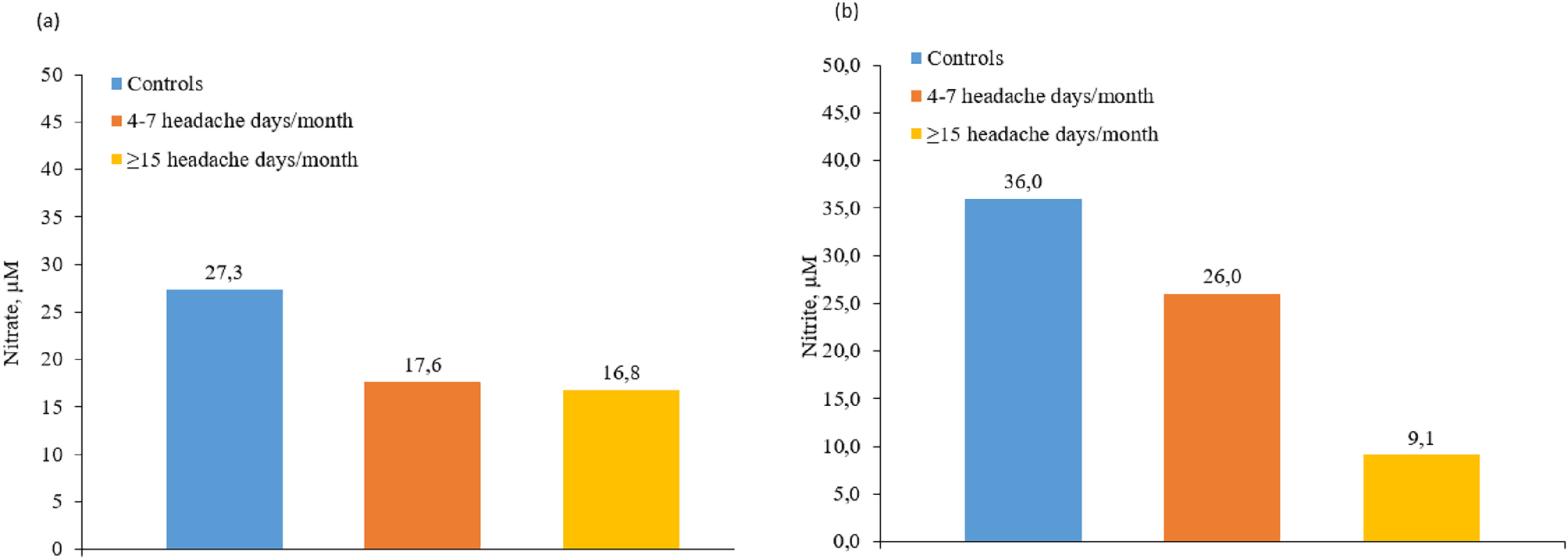
(**a**) Nitrate and (**b**) nitrite levels at baseline by migraine severity. Despite not finding statistically significant differences, an inverse correlation trend was observed in nitrates (*p* > 0.05) and nitrites (*p* > 0.05). Low [4–7 headache days/month], high [8–14 headache days/month] and chronic [≥ 15 headache days/month of which ≥ 8 days are days with a migraine].

**Figure 4:**
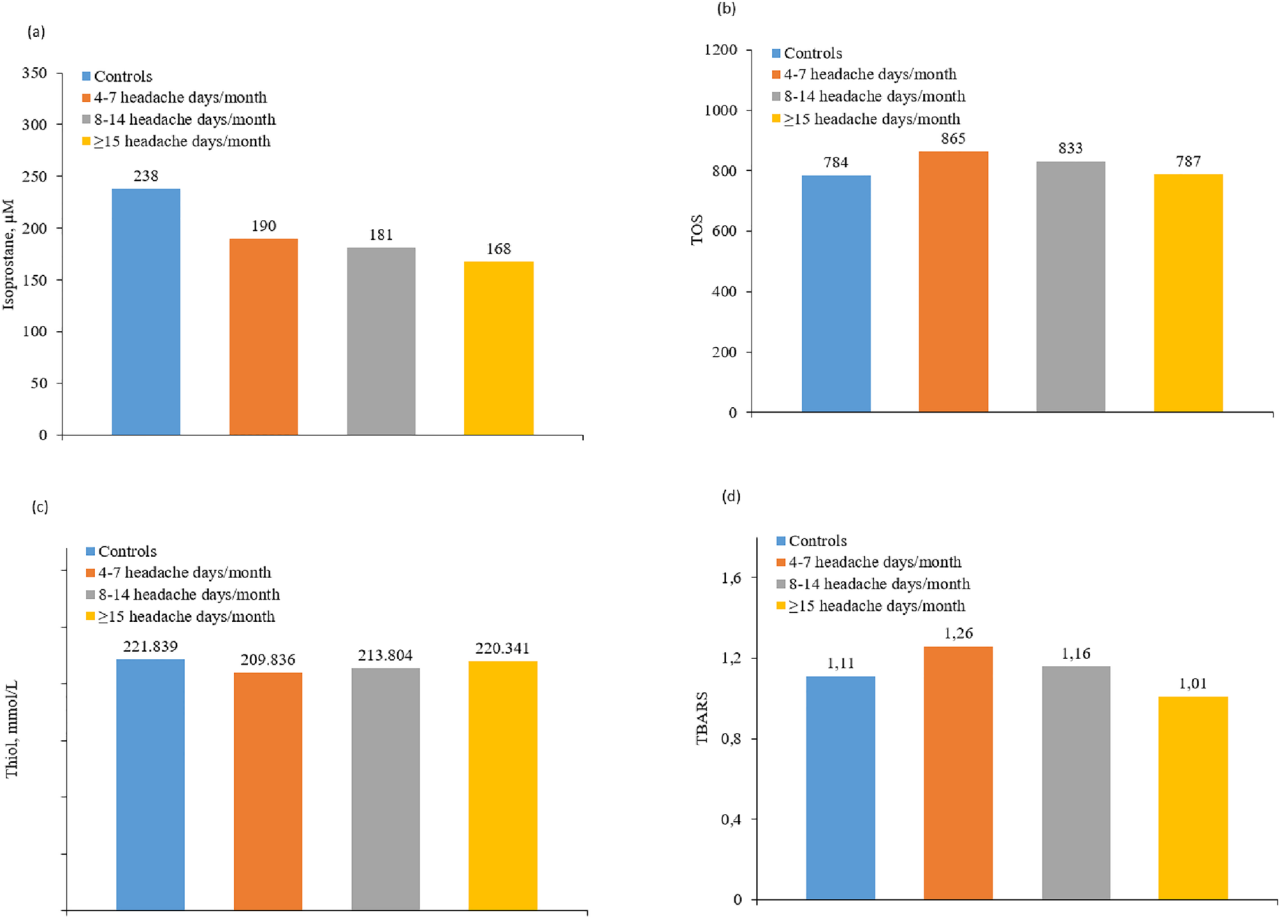
(**a**) Isoprostane, (**b**) TOS, (**c**) thiol, and (**d**) TBAR at baseline by migraine severity. Despite not finding statistically significant differences, an inverse correlation was observed in isoprostanes levels (*p* > 0.05). By contrast, TOS and TBARS were highest and thiols were lowest in the LFEM subgroup, and tended to normalize as migraine severity increased. Low [4–7 headache days/month], high [8–14 headache days/month] and chronic [≥ 15 headache days/month of which ≥ 8 days are days with a migraine]. *TBAR* thiobarbituric acid reactive substances, *TOS* total oxidant status.

A positive correlation was found between the inflammatory and endothelial dysfunction biomarkers: CIMT (0.54 mm in controls, 0.55 mm in LFEM, 0.56 mm in HFEM and 0.60 mm in CM; *p* = 0.024), vWF antigen (110 IU/dL in controls, 114 IU/dL in LFEM, 133 IU/dL in HFEM and 140 IU/dL in CM; *p* = 0.045), and vWF activity (90% in controls, 90.9% in LFEM, 111% in HFEM and 123% in CM; *p* = 0.013). In the case of hsCRP, we observe a trend, but it does not reach statistical significance (1.54 mg/dL in controls, 2.38 mg/dL in LFEM, 2.68 mg/dL in HFEM and 3.25 mg/dL in CM; *p* > 0.05) (Fig. 2). Despite not finding statistically significant differences, an inverse correlation was observed in nitrates (27.3 µM in controls, 17.6 µM in LFEM and 16.8 µM in CM; *p* > 0.05), nitrites (36 µM in controls, 26 µM in LFEM and 9.1 µM in CM; *p* > 0.05) (Fig. 3) and isoprostanes (238 µM in controls, 190 µM in LFEM, 181 µM in HFEM and 168 µM in CM; *p* > 0.05) (Fig. 4a) and migraine frequency subgroups were observed. By contrast, TOS and TBARS were highest and thiols were lowest in the LFEM subgroup, and tended to normalize as migraine severity increased (Fig. 4b–d).

#### Biomarkers by disease type

In the migraine group, with regard to aura status, participants with MA at T0 had higher levels of triglycerides (108 vs. 87 mg/dL; *p* = 0.032), platelets (293 vs. 255 mL/mm^3^; *p* = 0.037), and nitrite (14.7 vs. 8.71 µM; *p* = 0.015), and lower levels of HDL cholesterol (57.4 vs. 63.9 mg/dL; *p* = 0.045) than those with MO. There were no significant differences between the two disease types with regard to the other biomarkers assessed.

When comparing patients with the two different types of migraine (MA/MO) versus controls, on one hand, we found higher levels of triglycerides (108 vs. 84.1 mg/dL; *p* = 0.013), platelets (293 vs. 248 mL/mm^3^; *p* = 0.032), and nitrites (14.7 vs. 10.2 µM; *p* = 0.049), as well as lower levels of HDL cholesterol (57.4 vs. 66.8 mg/dL; *p* = 0.004) and isoprostanes (171 vs. 238 µM; *p* = 0.040) in patients with MA compared to controls without migraine.

On the other hand, we found significantly higher levels of hsCRP (2.49 vs. 1.64 mg/dL; *p* = 0.023) and lower levels of nitrates (18.4 vs. 27.3 µM; *p* = 0.042) in MO patients compared to controls without migraine.

#### Biomarkers by sex

In the migraine group, women had higher HDL cholesterol (63.1 vs. 51.4 mg/dL; *p* = 0.002), hsCRP (2.71 vs. 1.41 mg/dL; *p* = 0.002), platelets (274 vs. 229 mL/mm^3^; *p* = 0.011), fibrinogen (4.00 vs. 3.48 mg/dL; *p* = 0.003), plasminogen (112% vs. 101%; *p* = 0.026) and TOS (999 vs. 234; *p* < 0.001) at T0 compared with men.

On one hand, when comparing the group of women with migraine versus controls, we found that this group have higher levels of hsCRP (2.71 vs. 1.82 mg/dL; *p* = 0.049) and lower levels of HDL cholesterol (63.1 vs. 68.7, *p* = 0.047) than women without migraine. On the other hand, we found that men with migraine had higher levels of hsCRP (1.41 vs. 0.79, *p* = 0.045) than men without migraine, although, it is important to note that the sample size of this group is very small.

#### Prophylactic treatment response and change in biomarkers after treatment

During the prophylactic treatment period (3 months) 44/68 (64.7%) patients received metoprolol 50 mg/day while 24/68 (35.3%) received topiramate 25 mg/12 h.

After 3 months of prophylactic treatment (T1), there was a decrease from T0 in the mean number of headache days per month (from 9.3 to 3.0 days/month; *p* < 0.001). The majority of patients with migraine (61/68; 89.7%) responded to prophylactic treatment. 38/44 responded to metoprolol and 21/24 responded to topiramate.

At T1, when we compared the biomarkers between the patients who respond to the preventive treatment versus those who do not respond, we found that responders had significantly higher levels of nitrates (24.2–13.8 µM; *p* = 0.022) and nitrites (10.4–3.43 µM; *p* = 0.002) than non-responders. Moreover, while changes from T0 was observed in treatment responders in all endothelial dysfunction biomarkers investigated (Table 3), only theses inflammatory and endothelial dysfunction biomarkers were significantly changed: hsCRP levels decreased from 2.54 to 1.69 mg/dL (*p* = 0.045), vWF activity levels decrease from 124 to 103 IU/dL (*p* = 0.003), HDL cholesterol levels decreased from 61.7 to 55.5 mg/dL (*p* = 0.030) and prothrombin activity decreased from 1.01 to 0.93 (*p* = 0.01).

**Table 3:** Biomarkers in treatment-responsive patients with migraine at baseline and after 3 months of metoprolol/topiramate treatment.

	T0 (n = 88)	T1 (n = 61)	*p* value
Endothelial dysfunction biomarkers			
Total cholesterol, mg/dL	193 ± 34	185 ± 36.6	0.229
HDL cholesterol, mg/dL	61.3 ± 14.7	55.5 ± 12.2	**0.030**
LDL cholesterol, mg/dL	112 ± 28.5	111 ± 32.1	0.850
Triglycerides, mg/dL	105 ± 40.3	103 ± 51.9	0.843
hsCRP, mg/dL	2.54 ± 2.81	1.69 ± 1.64	**0.045**
Prothrombin activity, %	1.01 ± 0.10	0.93 ± 0.04	**0.010**
Antithrombin, %	106 ± 3.44	105 ± 12.3	0.573
Fibrinogen, mg/dL	3.94 ± 0.67	3.97 ± 0.61	0.782
Plasminogen, %	110 ± 17.4	106 ± 14.8	0.285
Factor II, %	106 ± 15.5	101 ± 13.1	0.150
Homocysteine, µmol/L	7.66 ± 2.76	8.89 ± 5.10	0.117
Platelet count, mL/mm^3^	269 ± 74.8	273 ± 81.6	0.741
vWF antigen, IU/dL	142 ± 20.2	124 ± 41.3	0.422
vWF activity, %	124 ± 4.03	103 ± 28.3	**0.003**
CIMT, mm	0.60 ± 0.12	0.55 ± 0.11	0.251
Nitrate, µM	19.1 ± 16.5	24.2 ± 20.0	0.183
Nitrite, µM	10.4 ± 9.69	12.3 ± 8.78	0.316
Isoprostane, µM	181 ± 134	215 ± 173	0.257
Oxidative stress biomarkers			
TOS, µmol/L	846 ± 653	852 ± 566	0.962
Thiol, nM	216.511 ± 33.763	215.119 ± 34.233	0.828
TBARS, µM	1.16 ± 0.55	1.17 ± 0.81	0.954

Significant values are in [bold].

All values are mean ± standard deviation.

*CIMT* carotid intima-media thickness, *hsCRP* high-sensitivity C-reactive protein, *HDL* high-density lipoprotein, *LDL* low-density lipoprotein, *PI* pulsatility index, *T0* baseline, *T1* 3 months post treatment initiation, *TBARS* thiobarbituric acid reactive substances, *TOS* total oxidant status, *vWF* von Willebrand factor.

No significant changes in the other biomarkers were observed after 3 months of prophylactic treatment; however, nitrate, isoprostane, and thiol levels were slightly increased and TOS and TBARS were slightly decreased from T0 to T1 (Table 3).

**Figure 5:**
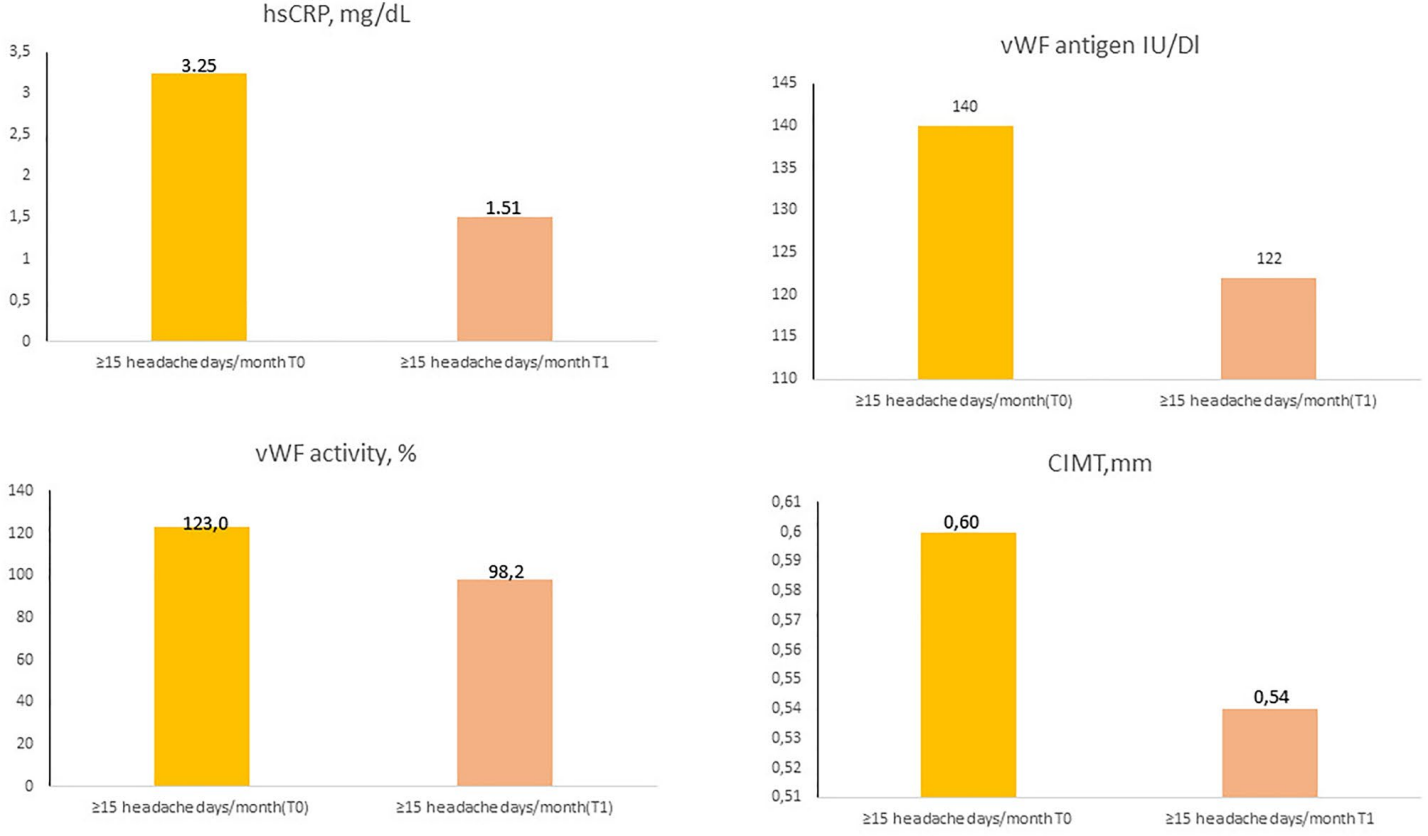
(**a**) hsCRP, (**b**) vWF antigen, (**c**) vWF activity, (**d**) CIMT comparation between T0 and T1 in CM patients [≥ 15 headache days/month of which ≥ 8 days are days with a migraine]. We observe differences in the following biomarkers: hsCRP decreased from 3.25 to 1.51 mg/dL (*p* > 0.05), vWF antigen decreased from 140 to 122 IU/dL (*p* > 0.05), vWFactivity decreased from 123 to 98.2 IU/dL (*p* > 0.05), CIMT decreased from 0.60 to 0.54 mm (*p* > 0.05). However, none of these differences reached statistical significance. *CIMT* carotid intima-media thickness, *hsCRP* high-sensitivity C-reactive protein, *vWF* von Willebrand factor.

**Figure 6:**
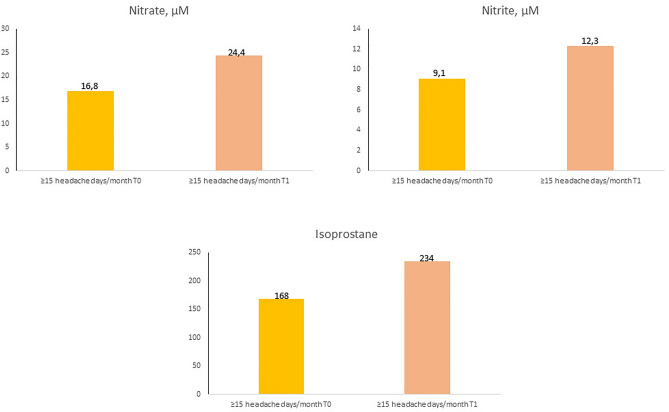
(**a**) Nitrate, (**b**) nitrite and (**c**) Isoprostane levels comparation between T0 and T1 in CM patients [≥ 15 headache days/month of which ≥ 8 days are days with a migraine]. Nitrate levels increased from16.8 to 24.4 µM (*p* > 0.05), nitrite levels increased from 9.12 to 12.3 µM (*p* > 0.05), and isoprostanes from 168 to 234 µM (*p* > 0.05). However, none reached statistical significance.

When we studied the effect of prophylactic treatment within different frequency subgroups, we observed that these differences at T1 were present across all subgroups, even though they only reach statistical significance in the case of hsCRP within the HFEM plus CM group (≥ 8 days migraine days/month), where levels decreased from 2.12 to 0.83 mg/dL (*p* = 0.048). Upon focusing on the CM subgroup alone, we found that hsCRP decreased from 3.25 to 1.51 mg/dL (*p* > 0.05), vWF antigen decreased from 140 to 122 IU/dL (*p* > 0.05), vWFactivity decreased from 123 to 98.2 IU/dL (*p* > 0.05), CIMT decreased from 0.60 to 0.54 mm (*p* > 0.05), while nitrate increased from 16.8 to 24.4 µM (*p* > 0.05), nitrite from 9.12 to 12.3 µM (*p* > 0.05), and isoprostanes from 168 to 234 µM (*p* > 0.05). None of these differences reached statistical significance (Figs. 5 and 6).

#### Change in biomarkers by prophylactic treatment received

After 3 months of prophylactic treatment, HDL cholesterol decreased significantly from baseline in patients with migraine who received metoprolol (from 61.2 to 54.2 mg/dL; *p* = 0.006). This was not observed in patients with migraine who received topiramate.

Direct comparison between the two treatments revealed higher thiol levels after 3 months in patients who received topiramate than in those who received metoprolol (229.7 vs. 207.6 mmol/L; *p* = 0.035).

## Discussion

The present study compared prophylactic treatment-naïve patients with episodic and CM to age/sex matched controls and found that patients with migraine, especially CM, had a pro-inflammatory state (higher hsCRP levels), an altered endothelial profile with lower levels of vasodilator factors (nitrate, nitrite, and isoprostanes), and worse vascular and pro-coagulant profiles (HDL cholesterol, vWF antigen and activity, and CIMT) compared with controls.

An association between hsCRP and migraine has been previously demonstrated in some case–control studies^26^. Elevated hsCRP levels in migraine have also been previously demonstrated during ictal or interictal periods^9,26,27^, suggesting that inflammation is present in both scenarios and might be a key factor in migraine pathophysiology, although no conclusive evidence of its relationship with aura status exists^8,26^. This pro-inflammatory state has also been associated with higher levels of vascular disease biomarkers insinuating a relationship between migraine and endothelial dysfunction^9,26,27^. Alternatively, some studies have found no differences in CRP levels between non naïve migraine patients and control groups^28^. This could be due to methodological differences: non-naive patients, patients with preventive and abortive treatment. In our study, despite the statistically significant differences, the hsCRP values remained within the normal limits both in the migraine and in the controls group.

There is no consistent evidence on whether vascular biomarkers such as cholesterol levels differ between migraine patients and healthy individuals^27^. Considering that migraines have been associated with an increased risk of stroke, mainly ischemic stroke^8,12^, and that ischemic stroke is related to cardiovascular risk, our findings of lower HDL cholesterol levels in patients with migraine compared with the control group, suggests an increased risk of vascular diseases in migraine. A recent metabolomics study proposed that patients with migraine have altered HDL cholesterol metabolism^29^. Another study found a positive association between metabolic syndrome and low HDL cholesterol in patients with MA^15^.

In the literature, the migraine-biomarker association appears to be influenced by migraine frequency and subtype, with MA demonstrating a stronger association than MO^10,30^. In the present study, significant differences in some biomarkers were observed between patients with MA and those with MO. A worse vascular profile was seen in patients with MA, with higher levels of triglycerides, platelets and lower levels of HDL cholesterol, compared with those with MO. No differences in hsCRP, vWF activity, or CIMT were seen across the two subtypes. On the other hand, some vasodilator factors (nitrite) were increased in patients with MA. This may have occurred due to a compensatory effect. We found a correlation between migraine frequency and biomarker levels, which are statistically significant in the case of CIMT, vWF antigen and vWF activity, with the most important biomarker alterations observed in patients with CM (independent of aura status), unlike findings from previous studies^31^.

We found vWF activity and CIMT significantly increased in the CM group compared with controls at T0. vWF is associated with cardiovascular risk factors as well as ischemic stroke^32^ and is a well-established marker of endothelial activation^27^. Xiang and colleagues suggest that vWF regulation may serve as a powerful therapeutic target in treating thrombotic diseases such as stroke or myocardial infarction^33^. However, differences in CIMT measurements in patients with migraine are conflicting. Wang and colleagues found that CIMT was increased in patients with migraine compared with controls, independent of aura status^34^. Other studies have found that patients with migraine and those with MA have elevated CIMT^30^. Our results support the idea that patients with migraine are likely to have vascular dysfunction, atherosclerosis and higher cardiovascular risk, these alterations are more serious in cases of more severe migraine. In contrast, some studies did not find any difference in CIMT levels between patients with migraine and controls^35^.

Regarding the influence of sex on biomarkers, we found a pro-inflammatory and pro-oxidative profile in women with migraine, with higher levels of hsCRP, fibrinogen, platelets and TOS in women compared with men, whereas HDL cholesterol levels were higher in women than in men, suggesting an atheroprotective effect and reduced vascular risk. However, comparisons between women and men in our study should be interpreted with caution, given the small sample size of men (n = 11).

We found statistically significant difference in endothelium-derived vasoactive factors between the migraine and control groups at T0 (nitrate in LFEM, nitrat and isoprostane in HFEM and CM group). Previous studies have found decreased interictal concentrations of nitric oxide metabolites in patients with migraine, especially in those with MA^9^. Endothelial dysfunction is characterized by a reduction in the bioavailability of vasodilators and the consequent impairment in the reactivity of vasculature. It is characterized by a decrease in bradykinin-mediated endothelial nitric oxide synthesis^9^. Isoprostanes play an important role in transducing the effects of metabolic and hemodynamic abnormalities on platelet and vascular smooth muscle cell activation. Increasing evidence suggest isoprostanes are involved in the complex interplay underlying cardiovascular homeostasis between low-grade inflammation, lipid peroxidation, and platelet activation^36^. A recent study found prostaglandin E2 (PGE2) serum levels were significantly lower in patients with migraines compared with controls. Moreover, low serum levels of PGE2 have been positively correlated with headache frequency^37^. With the present study, we wish to highlight that nitrates and isoprostanes may be useful biomarkers of migraine frequency and treatment response. Increasing evidence points towards the role of endothelial dysfunction in migraine.

Additionally, we observed higher levels of TOS and TBARS, and lower levels of thiol in patients with migraines, however, not significantly so. Previous studies provide evidence for the role of oxidative stress and altered metabolism in migraine pathophysiology, and highlight it as a suitable therapeutic target, although discrepancies do exist^38^. Gross and colleagues observed that one-third of patients with migraines had abnormally low thiol values^31^. Other studies have found serum TOS values to be significantly higher in patients with MO than in controls^36^. Togha and colleagues observed that patients with CM had lower total non-enzymatic antioxidant capacity (catalase, superoxide dismutase) and higher oxidative stress (nitric oxide, malondialdehyde) compared with patients with episodic migraines or healthy controls^39^.

In the present study, the differences between hsCRP, CIMT, vWF antigen, vWF activity, nitrite, nitrate and isoprostants levels had a positive correlation with migraine frequency (although it only reached statistical significance in the case of CIMT, vWF antigen, vWF activity). On the other hand, TBAR, TOS and thiol peaked in participants with LFEM (4–7 headache days per month) and normalized as migraine frequency increased. This may be due to a compensatory mechanism between pro- and antioxidant factors.

After 3 months of prophylactic treatment, when we compared the patients who responded to the preventive treatment with those who did not respond, we observed that responders had significantly higher levels of endothelium-derived vasoactive parameters (nitrates and nitrites) than non-responders. Moreover, at T1 all endothelial and oxidative parameters had improved in preventive treatment-responders patients, except for HDL cholesterol. HsCRP, vWF activity and prothrombin activity showed statistically significant improvements in this study. HDL cholesterol levels decreased in metoprolol recipients (and were unchanged in topiramate recipients); in both instances levels were < 60 mg/dL. Although HDL cholesterol levels ≥ 60 mg/dL are desirable in clinical practice, to date, no clinical trials have determined specific target levels. Of note, low HDL cholesterol levels are associated with residual risk in atherosclerotic cardiovascular disease patients^40^. Metoprolol has been associated with a decrease in HDL cholesterol in previous short and long-term studies. One hypothesis for this effect is that its indirect inhibition of lipoprotein lipase may lower HDL cholesterol plasma levels^41^. When we analyzed the impact of prophylactic treatment among various frequency subgroups, we noted that the differences in biomarker levels persisted, even though they did not achieve statistical significance. In the specific case of the CM group, we observed a decrease in hsCRP, vWF antigen, vWF activity, CIMT, and an increase in endothelium-derived vasoactive factors: nitrate, nitrite, and isoprostanes. However, these changes did not reach statistical significance. Nevertheless, when we examined the subgroup comprising HFEM plus CM (≥ 8 migraine days per month), we observed a statistically significant reduction in hsCRP levels. This finding could potentially be attributed to the limited sample size.

We observed topiramate/metoprolol treatment-related reductions in oxidative damage factors such as TOS and TBARS, and an increase in antioxidant activity (thiol) and vasodilator bioavailability (isoprostanes and nitrates). In our sample, thiol antioxidant activity was significantly higher in patients who had been treated with topiramate. In addition, metoprolol reduced HDL levels in a statistically significant way. Based on our results, it appears that topiramate may offer greater benefits as a first-line preventive treatment for patients with migraine. However, we cannot overlook the placebo effect of the drugs so further research is needed to corroborate these findings. Other migraine treatments have also been found to have antioxidant properties or limit oxidative stress, including flunarizine^19^, BoNT/A^20^, and anti-calcitonin gene-related peptide treatment^20^.

To the best of our knowledge, this is the first study that assesses both endothelial dysfunction and oxidative stress biomarkers after 3 months of prophylactic treatment in patients with episodic and CM. However, our study also has certain limitations. The study was not designed as a double-blinded, randomized clinical trial, and as a result, there is no data on adverse events and adherence to the medication regimen. Our sample size was relatively small. Moreover, despite the cases being consecutively enrolled, selection bias was possible. Although the studies were conducted at least 72 h after the intake of NSAIDs or other abortive treatments, we cannot guarantee that their anti-inflammatory effect might persist over time and interfere with the results. Moreover, factors such as diet and physical activity, which can alter oxidative stress levels, were uncontrolled and blood samples were not taken after night fasting. Additionally, the assessed biomarkers may be surrogate markers of changes in the brain. Another limitation may have been our short duration of follow-up, which may not have been long enough to detect other differences. It would be beneficial to increase the sample size, and the duration of follow-up (to 6 or 12 months), in future studies.

## Conclusions

Patients with migraines (MA or MO), especially CM, have differences in biomarker levels compared to controls suggesting endothelial and oxidative dysfunction. These patients also have a tendency to present with enhanced oxidative stress and lowered antioxidant activity during interictal periods. The differences in biomarker levels compared to controls are greatest among the migraine patients in the high-frequency and chronic migraine subgroups. Preventive migraine treatment for 3 months is capable of improving endothelial dysfunction biomarkers and modifying oxidative stress status even in patients with HEFM and CM. Based on these results, early initiation of a preventive treatment could reduce the risk of stroke events and other cardiovascular diseases. Topiramate would provide more benefits than metoloprol in prophylactic treatment- naïve patients with migraine. However, additional research is necessary to validate this information. The implications of a reduction in HDL cholesterol levels secondary to beta-blocker treatment in these patients remains to be determined.

### Supplementary Information

Supplementary Information 1.

Supplementary Information 2.

## Data Availability

The data supporting the conclusions of this article are available from the corresponding author upon reasonable request.

## Abbreviations

BoNT/A: Botulinum toxin A
CIMT: Carotid intima-media thickness
CM: Chronic migraine
EM: Episodic migraine
hsCRP: High-sensitivity C-reactive protein
HDL: High-density lipoprotein
HFEM: High-frequency episodic migraine
LFEM: Low-frequency episodic migraine
LDL: Low-density lipoprotein
MA: Migraine with aura
MO: Migraine without aura
PGE2: Prostaglandin E2
PI: Pulsatility index
SD: Standard deviation
T0: Baseline
T1: 3 Months post treatment initiation
TBARS: Thiol and thiobarbituric acid reactive substances
TOS: Total oxidant status
vWF: Von Willebrand factor
