# Supplementary material for: Prophylactic treatment can modify vascular risk biomarkers in high-frequency episodic and chronic migraine patients: a pilot study
Source: Sci Rep. 2023 Nov 8;13:19416. doi: 10.1038/s41598-023-44522-8 (PMC10632400; doi:10.1038/s41598-023-44522-8)
Supplement: Supplementary file 1 — Supplementary Information 1. [file 41598_2023_44522_MOESM1_ESM.docx]

**Supplementary Table S1.** Inclusion and exclusion criteria

|  | **Migraine group** | **Control group** |
| --- | --- | --- |
| **Inclusion criteria** | 1. Age 18–50 years | 1. Age 18–50 years |
|  | 1. Migraine diagnosis based on IHS criteria [1] | 1. No migraine diagnosis |
|  | 1. More than 4 migraine episodes per month |  |
|  | - 1. Low frequency (4–7 headache days per month) |  |
|  | - 1. High frequency (8–14 headache days per month) |  |
|  | - 1. Chronic migraine (≥15 headache days/month, of which ≥8 days are days with a migraine) |  |
|  | 1. No previous prophylactic treatment use |  |
| **Exclusion criteria** | 1. Presence of cardiovascular risk factors | 1. Presence of cardiovascular risk factors |
|  | 1. Presence of oncologic or inflammatory disease | 1. Presence of oncologic or inflammatory disease |
|  | 1. Smokers | 1. Smokers |
|  | 1. Use of antioxidant drugs | 1. Use of antioxidant drugs |
|  | 1. Use of pleiotropic drugs | 1. Use of pleiotropic drugs |
|  | 1. Use of drugs acting on the arterial wall | 1. Use of drugs acting on the arterial wall |
|  | 1. Use of hormonal contraceptive pills | 1. Use of hormonal contraceptive pills |
|  | 1. Pregnancy | 1. Pregnancy |
|  | 1. Previous use of any prophylactic migraine treatment |  |
| IHS, International Headache Society. | | |

**References**

1. International Headache Society (2018) Headache Classification Committee of the International Headache Society (IHS) The International Classification of Headache Disorders, 3rd edition. Cephalalgia 38(1):1-211. <https://doi.org/10.1177/0333102417738202>
