# Supplementary material for: Prophylactic treatment can modify vascular risk biomarkers in high-frequency episodic and chronic migraine patients: a pilot study
Source: Sci Rep. 2023 Nov 8;13:19416. doi: 10.1038/s41598-023-44522-8 (PMC10632400; doi:10.1038/s41598-023-44522-8)
Supplement: Supplementary file 2 — Supplementary Information 2. [file 41598_2023_44522_MOESM2_ESM.docx]

**Supplementary Table S2.** Biomarkers studied

| **Endothelial dysfunction biomarkers** | |
| --- | --- |
|  | Total, LDL and HDL cholesterol |
|  | Triglycerides |
|  | hsCRP |
|  | Prothrombin activity |
|  | Antithrombin |
|  | Fibrinogen |
|  | Plasminogen |
|  | Factor II |
|  | Homocysteine |
|  | Platelet count |
|  | vWF antigen and activity |
|  | CIMT - Ultrasonographic measurement; increased value has been considered an early marker of atherosclerosis [1] |
| 13.  14.  15. | PI - Ultrasonographic measurement assessing arterial stiffness, considered a predictor of comorbidity and mortality in cardiovascular diseases [1]  Nitrate/nitrite - Stable metabolites of nitric oxide; important vasodilators whose synthesis is impaired during oxidative stress. Defects in nitrate/nitrite production or activity has been linked to endothelial dysfunction [2]  Isoprostanes |
| **Oxidative stress biomarkers** | |
|  |  |
|  | TOS |
|  | Thiol - Serum thiols are strong antioxidants, physiologically free-radical scavengers and modulate anti-oxidant enzymes related to glutathione [3] |
|  | TBARS - End products of highly reactive and unstable lipid peroxides generated from membrane degradation; non-specific witness of oxidative stress [4] |
| CIMT, carotid intima-media thickness; hsCRP, high-sensitivity C-reactive protein; HDL, high-density lipoprotein; LDL, low-density lipoprotein; PI, pulsatility index; TOS, total oxidant status; TBARS, thiobarbituric acid reactive substances; vWF, von Willebrand factor. | |

**References**

1. Tietjen GE, Khubchandani J (2015) Vascular biomarkers in migraine. Cephalalgia 35:95-117. <https://doi.org/10.1177/0333102414544976>

2. Cayman Chemical Company. TBARS Assay Kit. 2017. Acessed from: <https://cdn.caymanchem.com/cdn/insert/10009055.pdf> Acessed: 30 May 2022

3. Jiménez Caballero PE, Muñoz Escudero F (2013) Peripheral endothelial function and arterial stiffness in patients with chronic migraine: a case-control study. J Headache Pain 14(1):8. <https://doi.org/10.1186/1129-2377-14-8>

4. Magalhães JE, Barros IML, Pedrosa RP, Sampaio Rocha-Filho PA (2019) Migraine and markers of carotid atherosclerosis in middle-aged women: a cross-sectional study. Headache 59:77-85. <https://doi.org/10.1111/head.13460>
